# The Shape of Uterine Contractions and Labor Progress in the Spontaneous Active Labor

**Published:** 2015-03

**Authors:** Samira Ebrahimzadeh Zagami, Nahid Golmakani, Seyyed Ali-Reza Saadatjoo, Nayyereh Ghomian, Behjat Baghbani

**Affiliations:** 1 Department of Midwifery, School of Nursing and Midwifery, Mashhad University of Medical Sciences, Mashhad, Iran;; 2 Department of Nursing, School of Nursing and Midwifery, Birjand University of Medical Science, Birjand, Iran;; 3 Department of Gynecology, Emam-Reza Hospital, Mashhad University of Medical Science, Mashhad, Iran;; 4 Omolbanin Hospital, Mashhad University of Medical Science, Mashhad, Iran

**Keywords:** Uterine contraction, Dystocia, First stage labor

## Abstract

**Background:**

Dystocia is the most common indication of primary cesarean section. The most common cause of dystocia is uterine dysfunction. In prolonged labor, more attention is usually paid to the fetus and pelvis rather than to the role of uterine contractions in a delivery. Therefore, we decided to determine the relationship between the labor progress and uterine contractions shapes**.**

**Methods:**

In this cross-sectional study, 200 primiparous women participated having a single pregnancy and cephalic presentation. Uterus contractions were recorded using electronic fetal monitoring at the beginning of the active phase of labor (dilatation 3-5 cm) for 30 min. Fall to rise (F:R) ratio was calculated by determining the duration of returning from a contraction peak to its baseline (fall) and the duration of the rise time from baseline to peak (rise) in two groups. The data were analyzed using t-test and Chi-square test.

**Results:**

In this study, 162 women had a normal delivery and 38 women had a cesarean (CS) delivery due to the lack of labor progress. The average F:R ratio was 1.13±0.193 seconds in the vaginal delivery group and 1.64±0.301 seconds in the CS group. This difference was statistically significant (P<0.001). The frequency of contractions in the vaginal delivery group was more than the CS group (P=0.008).

**Conclusion:**

Our findings demonstrated that uterine contractions shapes change; and F:R ratio was higher in the group that lacked labor progress. Therefore, contraction shapes can be used to predict the labor progress.

## Introduction

Dystocia is characterized by abnormal slow progress of ‘labor progress’.^1^ Abnormal progress of labor is a common event in each stage of labor.^2^ Dystocia is the most common indication for primary cesarean section.^3^ The most common cause of dystocia is inefficient uterine contraction.^1^ Most of the cesarean deliveries with cephalic presentation (68%) are due to the lack of labor progress.^3^^,^^4^

In Iran, cesarean rates were 48% in university hospitals and 90% in private hospitals in 2010,^5^ and the most common cause of cesarean is the fear of labor pain.^6^

Studies have shown that nulliparity is an important risk factor to the diagnosis of dystocia.^3^^,^^7^In primiparous women, in spite of adequate contractions, fetopelvic disproportion is diagnosed when slow or no labor progress occurs for 2-4 hours, and cervical dilatation is at least 3 cm or preferably 4 cm.^8^The main causes of prolonged labor can be due to the problems related to the pelvic, fetus and uterine contractions.^2^^,^^9^ The most common cause of poor labor progress is inadequate uterine contractions.^2^In dystocia, studies that were done about role of the fetus and pelvis is more than uterine contraction.^10^

Poor functioning of uterine may be due to its incomplete polarization. Studies on isolated myometrial tissue have shown that, there is a relationship between electrical events and myometrial contractions. Contraction and relaxation of myometrium occur as the result of periodic repolarization and depolarization of the muscle cell membranes.^11^ On the other hand, it has been found that uterine contractions display a characteristic pattern in cephalopelvic disproportion, and the duration of returning from a contraction’s peak to its baseline (fall) in comparison with the duration of rise time from baseline to peak (rise) is longer in dystocia than in normal vaginal deliveries.^12^^-^^14^ A new ratio was calculated, which is essentially greater in CPD labors than in regular labors. The average F:R ratio was 1.77 and 1.55 in the CS and vaginal delivery groups, respectively, indicating that the time of returning of each contraction from peak to baseline was longer in women with CS delivery.^10^ Pates et al. expressed that twelve or more contractions per hour in patients indicate more progress of cervical condition and fetal station, and consequently, cause the progress of labor.^15^ Oppenheimer demonstrated that, with the same level of uterine activity, when the number of contractions were 21 to 23, cervical dilatation reached to the maximum rate. In another study, in which primiparous and multiparous women were included, no relationship was observed between the number of contractions and cervical dilation.^16^

Technically, effective uterine contractions include three factors: intensity, synchronization, and frequency of contractions. Most studies are based on single-lead recordings that can reflect the severity and frequency of uterine contractions. Therefore, uterine synchronization topography can be used to display labor progress in the labor room.^17^ Prevention of the prolonged or arrested labor essentially depends on the rapid diagnosis of probable cephalopelvic disproportion. It is clear that poor and delayed progress in labor are associated with the increasing incidence of cesarean, labor augmentation, and the number of vaginal examinations.^18^ In most cases, diagnosis of cephalopelvic disproportion is retrospective. It means that, cephalopelvic disproportion is diagnosed when multiple interventions are done during a prolonged labor which do not lead to vaginal delivery.^10^ Many studies have been done in the field of prediction of cephalopelvic disproportion, including pelvimetry of pelvis, computed tomography and MRI (magnetic resonance imaging), to assess pelvic capacity and ultrasonic estimation of fetal weight.^19^^,^^20^ In addition, formulas based on fetus and pelvis measurements have been investigated, but unfortunately, the value of these parameters is poor as predictive criteria for cephalopelvic disproportion. According to the standards of care, women are encouraged to have vaginal delivery in the absence of vaginal delivery contraindications and macrosomia (>5000 g).^10^

Rapid diagnosis of abnormal labor progress and prevention of prolonged delivery significantly reduces the risk of postpartum hemorrhage, infection, arrested labor, uterine rupture, and related complications.^21^^,^^22^

Considering the importance of the fact that rapid diagnosis of cephalopelvic disproportion reduces mother’s pain and improves the management of successful delivery, we decided to study the relationship between labor progress and uterine contractions shape**s.**

## Materials and Methods

This cross-sectional study was conducted in the Omolbanin Hospital (Mashhad, Iran) during 2010-2011. The study was approved by the Research Council and Ethics Committee of Mashhad University of Medical Sciences. Institutional Review Board approval was obtained. After informing their consorts, 200 primiparous women were selected by convenience sampling. According to the method of Pokak and Althaus,^10^ the sample size was calculated to be 55 patients per group, but due to the limited number of women with cesarean delivery (38%) and due to the lack of progression in order to increase test throughput, the number of women with vaginal delivery increased to 162.

$$n=\frac{s_{1}^{2}+s_{2}^{2}}{{\left(m_{2}-m_{1}\right)}^{2}}f\left(\alpha \text{,}\beta \right)$$

$$\frac{{\left(\text{0.4}\right)}^{2}+{\left(\text{0.3}\right)}^{2}}{{\left(\text{1.77}-\text{1.55}\right)}^{2}}\times \text{10.5}=\text{54.23}≅\text{55}$$

The inclusion criteria were age (18-35 years), singleton pregnancy with cephalic term presentation, tendency to do vaginal delivery, having no medical or mental diseases, having no pregnancy complications, unrupture of membrane, no CPD in vaginal exam, and BMI<29. Exclusion criteria included: using oxytocin before and during the monitoring of contractions, performing cesarean section for any reason except for arrested delivery or pelvic constriction, the birth weight <2500 or >4000, and using the analgesia such as epidural anesthesia, morphine and pethidine during the study. Uterine contractions were recorded by using fetal monitoring system (Model FC1400) at the beginning of the spontaneous active labor (cervical dilatation 3-4 cm) for 30 min. The F:R ratio (second) was determined by measuring the time for a contraction to return to its baseline from its peak and the time for a contraction to rise to its peak. The F:R’s were averaged over the number of contractions. Then, the samples were practically set by amniotomy, use of oxytocin and hydration until the delivery time. Cesarean delivery was performed due to the lack of labor progress for more than two hours.

SPSS 11.5 software was used for data analysis. T test was used for comparison between the two groups and Chi-square test was used for categorical data. 

## Results

In total, 200 women participated in this study, of which, 162 patients had vaginal delivery, and 38 patients had cesarean (CS) delivery due to the lack of labor progress. Characteristics of the cases are shown in table 1.

**Table 1:** Comparison of demographic characteristics of the participants between the vaginal delivery and CS groups

**Demographic characteristics**	**Vaginal delivery (n=162)**	**Cesarean delivery (n=38)**	**t**	**P value**
	**M (SD)**	**M (SD)**		
Age (year)	22.6±3.007	25.68±4.55	5.069	<0.0001
Height (cm)	160.23±5.14	161.35±4.40	1.22	0.223
Weight (kg)	69.86±10.03	73.08±9.57	1.77	0.077

The mean age of participants in the vaginal delivery group was significantly less than the CS group (P<0.001) (table 1). There was no significant difference between the two groups in terms of BMI (P=0.93).

After monitoring, spontaneous rupture of the membranes was occurred in 42.2% of the cases and artificial rupture of the membrane was done for the rest of the cases (56.8%). 

After monitoring, in order to augment the labor, oxytocin was used in 29% of the CS group and in 24.2% of the vaginal delivery group. However, there was no significant difference between the two groups in this regard (P=0.54). Duration of the first stage of labor (since the admission in the active phase of labor until vaginal delivery or CS) was 5.19±2.7 hours in the vaginal delivery group and 8.13±3.1 hours in the CS group, and the difference was statistically significant (P<0.001).  

The frequency of contractions in 30 min. was significantly more in the vaginal delivery group than the CS group (P=0.002) (table 2). Also, the range of F:R ratio was 0.6-1.52 and 1-2.5 in the vaginal delivery and CS groups, respectively.

**Table 2:** Comparison of the pattern of contractions between the vaginal delivery and CS groups

**Characteristics of contractions pattern**	**Vaginal delivery (n=162)**	**Cesarean delivery (n=38)**	**t**	**P value**
	**M (SD)**	**M (SD)**		
Contraction frequency	8.3±2.30	7.05±1.46	3.192	0.002
F:R	1.13±0.193	1.64±0.301	9.981	<0.001

The F:R ratio was 1.13±0.193 in the vaginal delivery group and 1.64±0.301 in the CS group. This difference was statistically significant (P<0.001) (table 2) which indicates that in women with CS delivery, due to lack of labor progress, the return time of the contractions to baseline was longer in each contraction. Also a positive relationship was observed between the F:R ratio and the mode of delivery by using the Spearman’s correlation test (r=0.706, P<0.001). In women who did not receive oxytocin for labor augmentation, the frequency of contractions in 30 min. was 8.37±2.7 in the vaginal delivery group and 7.27±1.2 in the CS group, and there was no statistically significant difference between the two groups (P=0.07). On the other hand, in the women who received oxytocin for labor augmentation, the frequency of contractions was 7.92±1.6 in the vaginal delivery group and 7.22±1.9 in the CS group, and there was no statistically significant difference between the two groups (P=0.37).

The weight, height and head circumference mean in the CS group was significantly more than the vaginal delivery group (table 3). There was no significant difference in the sex of neonatal between the two groups (P=0.087).

**Table 3:** Comparison of the mean of weight, height and head circumference of the infants between the vaginal delivery and CS groups

**Infants** ** characteristics**	**Vaginal delivery (n=162)**	**Cesarean delivery (n=38)**	**t**	**P value**
	**M (SD)**	**M (SD)**		
Weight (g)	3211.55±309.63	3665.71±357.6	6.967	<0.001
Height (cm)	50.2±1.66	51.36±1.66	3.659	0.001
Head circumference (cm)	34.32±1.58	35.37±1.82	3.152	0.003

## Discussion

The results of this study showed that the shape of uterine contractions was different in the women who had a normal vaginal delivery than those with CS delivery due to the lack of labor progress and that the fall time of contractions was shorter in vaginal delivery. Similar to our findings, Althaus’s study observed that the F:R ratio was 1.77 in the CS group and 1.55 in the vaginal delivery group.^10^Vasak et al. showed that, in women with vaginal delivery with or without augmentation, the power density spectrum (PDS) peak frequency was significantly lower than women with cesarean section because of the first stage of the labor arrest.^23^Women who do not respond to augmentation seem to have higher PDS values in pre-augmentation which may be due to the presence of a certain degree of lactic acidosis.^23^

Increased numbers of K_ATP_ channels would hold the membrane potential close to K^+ ^balance value, which would negate membrane depolarization, which is essential for uterine contractions during the labor.^24^

The channels that hold recumbent membrane potential lead to membrane repolarization, which may be significant for holding uterine silence.^24^

Vasak et al. concluded that the PDS peak frequency of contractions which lead to cesarean section was significantly different from contraction in normal vaginal delivery.^23^

Fetal head station and cervical dilation are the two main physiological factors by which the progress of labor is evaluated, and uterine contractions affect both factors.^25^

It seems that the uterine activity is an adaptation mechanism, in case there is an obstacle during the fetal descent. It could be considered as a feedback mechanism in which the uterus receives the data of fetal descent progress and changes its ability accordingly. Successful adaptation enables the uterus to overcome the relative cephalopelvic disproportion and affect the mode of delivery.

In this study, there was a significant difference between the two groups in terms of mother’s age. A similar result was observed in the Althaus’s study. The F:R ratio was increased with increasing age, and there was a statistically significant difference between the two groups in this regard (P<0.001). One possible cause may be ageing-associated reduction in myometrial efficiency in older women.^24^ In Althaus’s study, although induction was done in 45% of the vaginal delivery group and in 65% of the CS group, the F:R ratio was higher in the CS group (1.76) than the vaginal delivery group (1.53) (P=0.003).^10^ Also, in the cases with spontaneous labor, the F:R ratio was higher in the CS group (1.81) than in the vaginal delivery group (1.5) (P<0.001).^10^ In this study, the samples were selected based on the spontaneous starting of the labor pain and no oxytocin was used for labor augmentation before recording the strip. After monitoring, oxytocin was used in 25.3% of the cases for labor augmentation. There was no significant difference between the two groups in this regard (P=0.54). Vasak et al. concluded that PDS values increased when using oxytocin.^23^

In addition, it was shown that the use of oxytocin in order to augment uterine contraction does not alter the shape of uterine contractions.^26^

In this study, the membranes were intact when the strips were recorded. Amniotomy was done if no spontaneous rupture of the membranes occurred until the delivery, and the fetal head was fixed in mother’s pelvis. Amniotomy was done in the CS group more than the vaginal delivery group (P=0.007). However, Althaus’s study showed that the rate of rupture of membrane did not have a significant difference in both groups (77% and 88% in vaginal delivery group and CS group, respectively, P=0.132).^10^

The aim of this study was to evaluate the labor progress and shapes of uterine contractions with spontaneous onset of labor. Therefore, it is recommended to study the shape of uterine contractions in the induction of labor. In addition, as the prevention of the first CS is one of the challenges in current obstetrics, and in this study, all of the CS’s were performed due to lack of progress labor. Therefore, the shape of uterine contraction in other causes of CS’s such as malpresentation, CPD and etc. are evaluated.  

The limitation of this study was the small number of women with CS due to the first stage of labor arrest. Therefore, it is recommended that a large cohort study to be conducted. 

## Conclusion

Our findings also showed that in the lack of labor progress, the shape of contractions changes, and the return to baseline of contraction becomes slower. Therefore, in addition to fetal and pelvic factors, considering the shape of contraction seems necessary in the prediction of labor progress. More studies are required to evaluate the relationship between the progress of labor, feedback mechanisms, and changes in the shape and pattern of contractions associated with the use of oxytocin and amniotomy. 
